# Distribution and ecological risk assessment of PEDCs in the water, sediment and *Carex cinerascens* of Poyang Lake wetland, China

**DOI:** 10.1038/s41598-019-47864-4

**Published:** 2019-08-05

**Authors:** Pinghua Yang

**Affiliations:** 1 College of Chemistry and Environment Engineering, Jiujiang University, Jiujiang, 332005 China; 2 Jiangxi Province Engineering Research Center of Ecological Chemical Industry, Jiujiang, 332005 China

**Keywords:** Environmental chemistry, Environmental impact

## Abstract

Phenolic endocrine disrupting chemicals (PEDCs), such as 4-nonylphenol (NP), 4-t-octylphenol (OP), bisphenol A (BPA), and nonylphenol-di-ethoxylate (NP2EO), can cause feminization and carcinogenesis. This study assessed the distributions of NP, OP, BPA, and NP2EO in the water, sediment, and *Carex cinerascens* of Poyang Lake wetland. The four PEDCs were ubiquitous. The concentrations of NP and OP in the water and sediment of the wetland were significantly lower than those in other regions of China. Average BPA concentrations in the water, sediment, and *Carex cinerascens* samples were 40.49 ± 18.42 ng/L, 9.840 ± 3.149 ng/g, and 3.25 ± 1.40 ng/g, respectively; the BPA concentration in the water was similar to that of other rivers in China. Average NP2EO concentrations in the wetland were 3125.9 ± 478.1 ng/L, 650.0 ± 209.9 ng/g, and 275.8 ± 59.0 ng/g in the water, sediment, and *Carex cinerascens* samples, respectively. The predicted no-effect concentrations in sediment for NP, OP, BPA, and NP2EO were estimated to be 75.41, 45.25, 8.22, and 237.5 ng/g, respectively. The risk quotient (RQ) method was used to characterise the ecological risk from these PEDCs. A high ecological risk (RQ ≥ 1) from BPA was observed for 0%, 57.69%, and 5.00% of water, sediment, and *C. cinerascens* samples, respectively, while a high risk from NP2EO was observed for 71.43%, 96.15%, and 55.00% of samples. Ecological risk varied spatially. The high ecological risk from NP2EO in Poyang Lake wetland may be a result of non-point pollution from rural areas and sewage from Poyang Lake basin.

## Introduction

Phenolic endocrine disrupting chemicals (PEDCs) are phenolic substances derived mainly from non-ionic surfactants that are widely used in industrial and household products such as detergents, emulsifiers, solubilisers, and dispersing agents^1–4^. PEDCs include 4-nonylphenol (NP), 4-t-octylphenol (OP), bisphenol A (BPA), and nonylphenol-di-ethoxylate (NP2EO). PEDCs interfere with the secretion, synthesis, metabolism, transportation, reactions, combination, and elimination of natural hormones in organisms. Due to their persistence, lipophilicity, toxicity, and endocrine disruption, PEDCs have harmful effects on various organisms, including feminization and carcinogenesis^5–10^. The wide distribution of PEDCs in the natural environment, and the associated adverse effects on wildlife, necessitate assessment of their distribution in ecosystems.

In China, there are many ecological and human health risk assessments for PEDCs. These studies include assessments of estrogens in the water, sediment, and biota of northern Lake Taihu^11^; NP and BPA in the Cape D’Aguilar Marine Reserve, Hong Kong^12^; APs, BPA, and TBBPA in Lake Taihu^13^; bisphenol analogues in the water and sediment of Liaohe River Basin and Lake Taihu^14^; EDCs in the Pearl River^15^; NP in Jiaozhou Bay in Qingdao^16^; estrogens and BPA in arid and semiarid areas of northwest China^17^; bisphenol analogues in the surface water and sediment of shallow freshwater lakes^18^; NP and OP in the riverine waters and surface sediments of the Pearl River Estuary^19^; 50 phenolic compounds in the surface water, sediment, and suspended particulate matter of three important rivers in Tianjin^20^; and eight EDCs of Huai River^21^. To date, however, there has been no study of the ecological risk from PEDCs in Poyang Lake—the largest freshwater lake in China.

**Figure 1:**
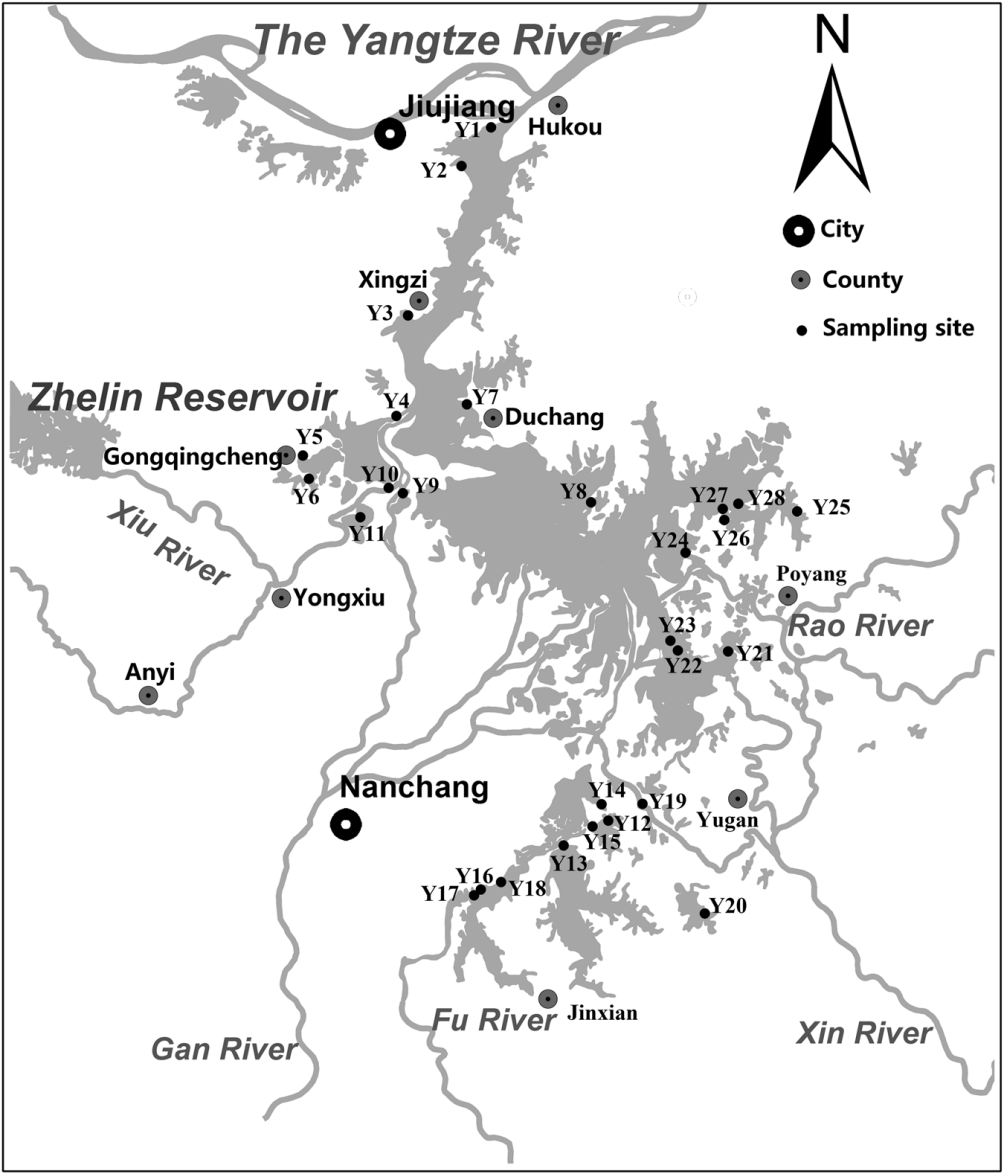
Sampling sites in Poyang Lake wetland, China.

Poyang Lake (115°49′–116°46′E and 28°24′–29°46′N; Fig. 1) is extremely important to migratory birds and regional ecological security. Poyang Lake is a typical shallow lake of eastern China, developed on alluvial plains with high-nutrient sediments. The area of Poyang Lake basin is 162 200 km^2^, which is 97% of the area of Jiangxi Province and its water level fluctuates considerably between the dry (from October to March) and wet seasons (from April to September). Poyang Lake is the only member of the International Living Lakes Network in China and is one of the most important ecological zones in the world (http://www.jxpoyanglake.gov.cn). The wetland is the largest winter migratory bird habitat in East Asia, hosting more than 500,000 migratory birds belonging to 105 species in 2016 and more than 95% and 80% of the global *Grus leucogeranus* and *Ciconia boyciana* populations, respectively. *Carex cinerascens* is the dominant species in the Poyang Lake wetland, with coverage between 63.27–100%. This species provides good wild pasture for cattle, sheep, and geese and its roots provide habitat for vivipara, mussels, and other food favorites of migratory birds. The lake has unique hydrological characteristics and colloid behavior; most colloids overflow into the Yangtze River, with short hydraulic residence times^22,23^. There are 18 protected areas in Poyang Lake, with a total area of 2006.18 km^2^ designated for migratory birds and wetland ecosystem protection. The lake is an important reservoir in the main stream of the Yangtze River, and plays an important role in regulating floods and protecting biodiversity in the Yangtze River Basin.

The generic framework and guidelines of the US Environmental Protection Agency (EPA) propose the following process for ecological risk assessments: (1) derive the predicted no-effect concentration (PNEC), and (2) calculate the risk quotient (RQ), which is the ratio of the predicted environmental concentration (PEC) to the PNEC^24^. In the technical guidelines for risk assessments formulated by the European Commission, the allocation balance formula can be used to calculate the predicted no-effect concentration in sediment (PNEC_sediment_)^25^. There are several reports of the PNEC or water quality criteria for PEDCs. Four species sensitivity distribution (SSD) models were used to calculate the PNEC for BPA^26^. The PNEC values for nonylphenol were also derived using SSD models and these were used to assess its ecological risk in the coastal waters of China^27^. The PNEC for BPA was derived from both acute and chronic toxicity endpoints using the SSD model and was then used to assess the ecological risk from BPA in surface waters of China^28^. Both maximum and continuous concentration criteria were derived for nonylphenol and used to assess its ecological risk to aquatic life in Chinese surface freshwater^29^.

This study aimed to investigate the distributions of NP, OP, BPA, and NP2EO in the water, sediment, and *Carex cinerascens* of Poyang Lake and use these to assess the ecological risk. The SSD model was used to estimate the HC_5_ and PNEC_water_ values for NP, OP, BPA, and NP2EO. According to the distribution equilibrium method (from the technical guidance document of the EU), the PNEC_sediment_ values of NP, OP, BPA, and NP2EO were estimated and used to assess the ecological risk from these four PEDCs.

## Results and Discussion

### Distributions of NP, OP, BPA, and NP2EO in the water, sediment, and *Carex cinerascens* of Poyang Lake wetland

The concentrations of NP, OP, BPA, and NP2EO in water, sediment and *Carex cinerascens* samples from twenty-eight sampling sites of the Poyang Lake wetland are shown in Table 1. For comparison, the range (mean) concentrations of NP, OP, and BPA in the surface waters and sediment of lakes and rivers of China are shown in Supplementary Information Table S9. All four PEDCs were ubiquitous. Average NP concentrations in the water, sediment, and *Carex cinerascens* samples were 1.468 ± 0.531 ng/L, 2.247 ± 1.273 ng/g, and 0.504 ± 0.178 ng/g, respectively. Average OP concentrations were 2.967 ± 1.409 ng/L, 2.247 ± 1.273 ng/g, and 0.201 ± 0.097 ng/g, respectively and average BPA concentrations were 40.49 ± 18.42 ng/L, 9.840 ± 3.149 ng/g, and 3.25 ± 1.40 ng/g, respectively. The concentrations of NP and OP in the water of the Poyang Lake wetland were significantly lower than those in Lake Taihu, Dianchi Lake, and other rivers in China^13,17,30–33^. This may be because some pollutants are removed by exchanged water and the water exchange speed is higher in Poyang Lake than in Lake Taihu and Dianchi Lake^22,34^. The BPA concentration in the water of the Poyang Lake wetland was similar to that of Lake Taihu, Dianchi Lake, and other rivers in China. Notably, the concentrations of NP, OP, and BPA in the waters of the Pearl River Delta region were found to be significantly higher than those in other regions of China, which may be attributable to the Pearl River coast being the most economically developed area in China, with the largest population density and high consumption of detergents^15,19,30^. The concentrations of NP, OP, and BPA in the sediment of Poyang Lake wetland were significantly lower than those in other regions of China. Average NP2EO concentrations in the wetland were 3125.9 ± 478.1 ng/L, 650.0 ± 209.9 ng/g, and 275.8 ± 59.0 ng/g in the water, sediment, and *Carex cinerascens* samples, respectively. Due to the high water-exchange speed, the concentrations of NP2EO, the primary degradation product of detergent, were significantly higher than those of NP, the advanced degradation product.

**Table 1:** Concentrations of 4-nonylphenol (NP), 4-t-octylphenol (OP), bisphenol A (BPA), and nonylphenol-di-ethoxylate (NP2EO) in surface water, sediment, and *Carex cinerascens* samples from the Poyang Lake wetland in the dry season (October to February).

Sites	Water (ng/L)				Sediment (ng/g dw)				*Carex cinerascens* (ng/g dw)			
	NP	OP	BPA	NP2EO	NP	OP	BPA	NP2EO	NP	OP	BPA	NP2EO
Y1	1.359	1.819	21.58	3028.2	2.03	0.665	9.058	483.5	0.544	0.036	2.42	233.6
Y2	2.585	2.237	22.21	3818.7	1.534	0.774	6.069	321.3	0.169	0.062	0.4	121.1
Y3	1.327	1.290	23.07	3651.0	2.683	3.578	14.005	422.8	0.607	0.134	3.39	188.6
Y4	7.387	21.241	25.81	3410.2	2.278	1.028	11.101	410	2.19	0.16	3.3	104.4
Y5	0.362	0.480	25.6	2333.4	1.922	4.237	9.288	369.3	0.446	0.072	4.54	241.3
Y6	2.223	1.831	37.03	4411.6	2.325	1.43	5.712	1063.9	0.516	0.066	2.54	206.4
Y7	1.207	0.887	24.97	2653.0	3.535	5.327	2.249	3107.7	0.809	0.115	4.22	273.1
Y8	2.281	1.160	25.74	3434.3	3.579	0.507	4.378	1173.5	0.541	0.085	3.01	244.6
Y9	1.751	1.929	22.98	3617.0	1.551	0.193	2.615	598.5	0.366	1.22	2.09	801.9
Y10	1.692	2.140	21.58	4242.4	0.126	0.504	15.779	127.2	0.229	0.313	4.14	311.5
Y11	1.183	1.587	17.21	3617.0	2.422	0.745	13.75	576.9	0.257	0.106	4.39	332.4
Y12	3.782	5.075	28.98	162.1	2.759	1.631	9.637	563.2	0.22	0.159	4.3	406.4
Y13	1.852	0.875	18.51	2750.2	—	—	—	—	—	—	—	—
Y14	1.297	1.450	19.97	3048.2	2.081	1.142	21.415	530.9	0.328	0.082	4.09	340.2
Y15	0.672	1.717	34.55	3023.4	—	—	—	—	—	—	—	—
Y16	1.038	1.618	48.59	1532.5	0.667	1.148	9.5	403.7	—	—	—	—
Y17	0.337	1.448	27.85	2374.2	0.717	1.367	3.025	503	0.308	0.304	17.37	353.8
Y18	0.746	4.548	66.86	4473.0	0.216	1.749	1.328	545.6	1.1	0.147	1.02	131.5
Y19	2.002	3.832	45.41	4182.0	0.548	1.051	1.485	306.9	0.424	0.136	0.55	121.2
Y20	0.059	1.093	177.6	850.2	0.724	1.822	1.688	600.7	—	—	—	—
Y21	0.442	2.526	21.69	1698.3	0.158	1.106	38.38	436.3	0.421	0.188	0.45	206.1
Y22	0.903	3.534	32.27	3909.0	1.159	1.232	18.69	495	—	—	—	—
Y23	0.037	0.143	3.84	421.8	1.115	1.523	11.07	600.1	0.048	0.147	0.38	210.9
Y24	0.944	3.206	242.2	4485.0	0.818	1.326	4.068	574.5	0.323	0.232	1.15	394.9
Y25	0.744	2.644	17.66	2878.2	0.235	1.502	12.43	626.8	—	—	—	—
Y26	0.480	5.540	45.87	5574.0	1.707	1.508	9.352	814.4	—	—	—	—
Y27	0.534	4.526	9.60	4770.0	3.98	1.226	17.39	515.5	0.234	0.258	1.15	292.9
Y28	1.883	2.706	24.57	3177.0	17.56	3.672	2.385	728.7	—	—	—	—
mean± SD	1.468± 0.531	2.967± 1.409	40.49± 18.42	3125.9± 478.1	2.247± 1.273	1.615± 0.471	9.840± 3.149	650.0± 209.9	0.504± 0.178	0.201± 0.097	3.25± 1.40	275.8± 59.0

Line segment (—) indicates that no samples were collected at this site. dw = dry weight.

### SSD curves and PNEC values

**Figure 2:**
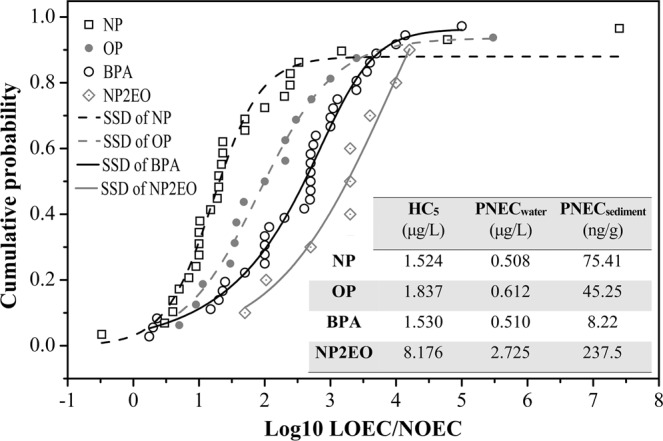
The species sensitivity distribution (SSD) curves for exposure concentrations of 4-nonylphenol (NP), 4-t-octylphenol (OP), bisphenol A (BPA), and nonylphenol-di-ethoxylate (NP2EO) in freshwater.

Using the chronic toxicity data for NP, OP, BPA, and NP2EO (S1 to S4), SSD models were constructed with a sigmoid distribution and the simulated curves for NP, OP, BPA, and NP2EO for freshwater ecosystems are shown in Fig. 2. The HC_5_ values of NP, OP, BPA, and NP2EO for freshwater ecosystems were estimated according to the constructed SSD curves (shown in Supplementary Information Table S10). The PNEC_water_ values of NP, OP, BPA, and NP2EO were calculated using the HC_5_ values, and these are shown in Fig. 2. In this study, the HC_5_ value of NP in water (1.524 μg/L) was similar to that in the literature (1.43 μg/L)^27^. This may be due to the adoption of similar SSD and data fitting methods. The PNEC_water_ of NP (0.508 μg/L) was slightly higher than that in the literature (0.33 μg/L)^35^ and the PNEC_water_ of BPA (0.510 μg/L) was lower than that in the literature (0.86 μg/L)^28^. These results may be due to differences in the chronic toxicological data used; thirty-five samples were used in this study, compared with ten in previous studies. The PNEC_water_ of NP2EO (2.725 μg/L) was 25 times higher than the 0.11 μg/L estimated from LC_50_ divided by the extrapolation factor^36^. The PNEC_sediment_ of NP2EO (0.2375 μg/g) was similar to that in the literature (0.25 mg/kg)^37^.

The SSD methodology requires the least amount of data in order to reliably estimate the PNEC. The OECD recommends a minimum of five no observed effect concentrations (NOECs)^38^, while the EU’s technical guidance document requires at least ten from species of at least eight taxonomic groups^25^. The data in this study meet the above requirements and provide a reference for ecological risk assessment. To date, there has been no report on the ecological risk from NP, OP, BPA, and NP2EO in *Carex cinerascens*. In this study, the HC_5_ and PNEC_sediment_ values were used to assess the ecological risk from NP, OP, BPA, and NP2EO to *Carex cinerascens*.

### Ecological risk assessment of NP, OP, BPA, and NP2EO in Poyang Lake wetland

The RQ values of NP, OP, BPA, and NP2EO in the water, sediment, and *Carex cinerascens* of Poyang Lake wetland are listed in Supplementary Information Table S11. The RQ values of NP and OP for all sampling sites were significantly lower than 1.0 and lower than those of other regions in China, indicating a low ecological risk from NP and OP^13,19,27^.

**Figure 3:**
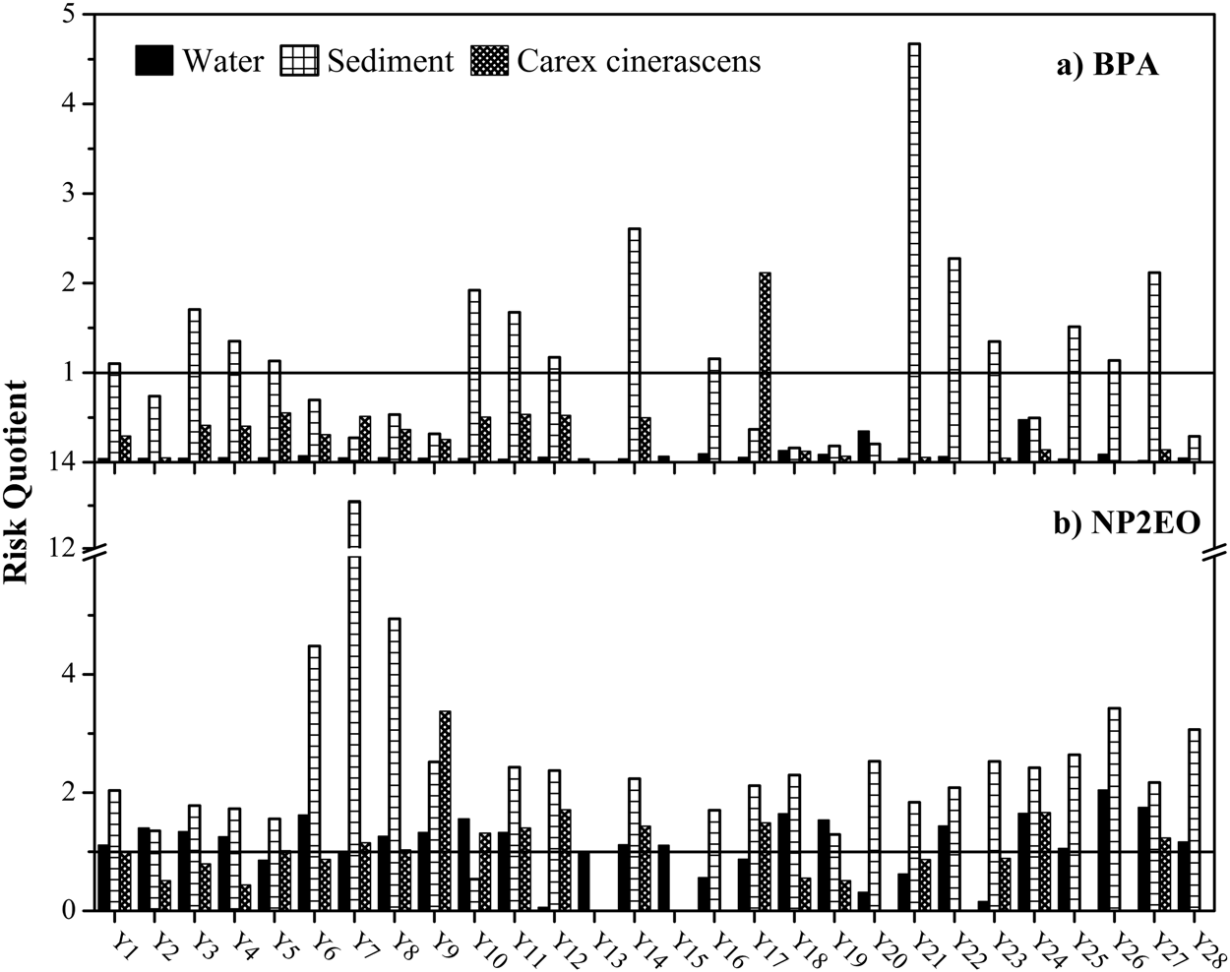
Risk quotient (RQ) values of bisphenol A (BPA) and nonylphenol-di-ethoxylate (NP2EO) in the water, sediment, and *Carex cinerascens* of Poyang Lake wetland.

The ecological risk from BPA and NP2EO in the water, sediment, and *Carex cinerascens* of Poyang Lake wetland is shown in Fig. 3; the horizontal line at RQ = 1.0 indicates high ecological risk. The RQ values of BPA in the water ranged from 0.0076 to 0.4751, with an average of 0.0794, and 10.71% of sites showed medium ecological risk. The RQ values of NP2EO in the water ranged from 0.059 to 2.045, with an average of 1.147. The ecological risk from NP2EO was medium and high at 25.00% and 71.43% of sites, respectively. The RQ values of BPA in the sediment ranged from 0.161 to 4.671, with an average of 1.198, and 42.31% and 57.69% of sites showed medium and high ecological risk, respectively. The RQ values of NP2EO in the sediment showed that 96.15% of sites had high ecological risk; values ranged from 0.536 to 13.09, with an average of 2.737. The RQ values of BPA and NP2EO in *Carex cinerascens*, showed that 75.00% and 5.00%, and 45.00% and 55.00% of sites showed medium and high ecological risk, respectively. In the water, sediment, and *Carex cinerascens* of Poyang Lake, there is potential ecological risk from BPA and NP2EO. The ecological risk from BPA in the water and sediment was similar to that in Lake Taihu^11,13,28^.

### Spatial ecological risk of Poyang Lake wetland

**Figure 4:**
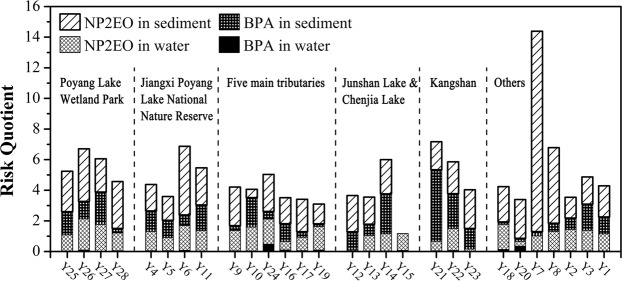
Spatial risk quotient (RQ) values of bisphenol A (BPA) and nonylphenol-di-ethoxylate (NP2EO) in the water and sediment of Poyang Lake.

The spatial RQ values for BPA and NP2EO in the water and sediment of Poyang Lake wetland are shown in Fig. 4. The ecological risk from BPA in Gan River (Y9), Xiu River (Y10), Fu River (Y16), Xin River (Y19), and Rao River (Y24)—the five main tributaries of Poyang Lake—was low in water and high in sediment; the ecological risk of NP2EO was high in both water (except for Fu River) and sediment (except Xiu River). The average RQ values were 1.25 for NP2EO in water, 0.74 for BPA in sediment, and 1.764 for NP2EO in sediment, indicating high ecological risk for input water to Poyang Lake. In Poyang Lake Wetland Park (Y25-Y28), the ecological risk from BPA was low in water and high in sediment, except for Baishazhou (Y28); the ecological risk from NP2EO was high in both water and sediment. The average RQ values were 1.504 for NP2EO in water, 1.264 for BPA in sediment, and 2.827 for NP2EO in sediment. Surrounding non-point pollution from rural areas and sewage wastewater are the likely cause for the high ecological risk.

In Jiangxi Poyang Lake National Nature Reserve (Y4-Y6, Y11), the ecological risk from BPA was low in water and high in sediment, except for in South Lake (Y6); the ecological risk from NP2EO was high in water and sediment, except for in Gongqingcheng (Y5). The average RQ values were 1.264 for NP2EO in water, 1.212 for BPA in sediment, and 2.548 for NP2EO in sediment. The high ecological risk may be due to the location of Jiangxi Poyang Lake National Nature Reserve at the convergence of the main branches of the Gan and Xiu Rivers; the reserve is also surrounded by Nanchang (which had an urban population exceeding 2 million in 2018, http://www.nc.gov.cn/), Anyi, Yongxiu, Gongqingcheng, De’an, and other cities. In Junshan Lake and Chenjia Lake (Y12-Y15), the ecological risk from BPA was low in water and high in sediment, except for in Junshan Lake (Y13); the ecological risk from NP2EO was high in both water (except in Sanlixiang (Y12)) and sediment. The average RQ values were 0.82 for NP2EO in water, 1.506 for BPA in sediment, and 2.124 for NP2EO in sediment. In the Kangshan area (Y21-Y23), BPA and NP2EO in the sediment were at high ecological risk levels, with averages of 2.764 and 2.149, respectively. Construction of dykes can alter hydrologic factors such that water exchange times are longer and water flow is slower. This may explain the high ecological risk from NP2EO in Zhu Lake, Junshan Lake, Chenjia Lake, and Kangshan, which are isolated from Poyang Lake by dykes. The particularly high ecological risk from NP2EO (RQ = 13.09) at Jishan (Y7) may be related to its proximity to Duchang city. Similarly, Xingzi (Y3) is also located close to Xingzi city, resulting in high ecological risk (except for BPA in water). Although the concentrations of NP and OP in water, and NP in *Carex cinerascens* at Liaonan (Y4) were higher than those at other sites, they were still lower than those of other lakes in China^13,32^ and the ecological risk was still very low. The concentrations of NP2EO in the water of Sanlixiang (Y12) and NP in the sediment of Baishazhou (Y28) were similar. The ecological risk from NP2EO in Gan River (Y9) was high, which may be attributable to the high consumption of detergents near the Gan River coast, the most developed area in Jiangxi province. The high ecological risk from BPA in Carex cinerascens from the Fu River Tributary (Y17) may be due to the fact that there are some small plastic factories upstream (http://www.ncx.gov.cn/articles/2019/05/14/577160.shtml), however, the risk was still lower than in other lakes of China^13,14,32,39^. The high ecological risk from NP2EO may be due to the fact that the Poyang Lake basin (162 200 km^2^) covers 97% of the area of Jiangxi Province, and sewage produced by the consumption of household products by 46.2 million people (2017), converges in Poyang Lake (http://www.jiangxi.gov.cn/). In the water, sediment, and *Carex cinerascens* of Poyang Lake wetland, the high ecological risk from BPA and NP2EO may cause harm to some organisms, especially migratory birds, which warrants further investigation.

## Conclusions

The distributions of NP, OP, BPA, and NP2EO in the water, sediment, and *Carex cinerascens* of Poyang Lake and their ecological risk were investigated. The four PEDCs were ubiquitous. This study adopted the SSD methodology to derive PNEC_water_ and PNEC_sediment_ values for NP, OP, BPA, and NP2EO. The RQ method was used to quantify ecological risk. The ecological risk from NP and OP in the water, sediment, and *Carex cinerascens* from all sampling sites was low. The ecological risk from BPA was low in the water and high in the sediment and *Carex cinerascens*, while that of NP2EO was high in the water, sediment, and *Carex cinerascens*. The ecological risks from BPA and NP2EO in the five main tributaries of Poyang Lake were high indicating a high ecological risk in the input waters to Poyang Lake. In Poyang Lake Wetland Park, Jiangxi Poyang Lake National Nature Reserve, and other lakes, the ecological risks from BPA and NP2EO were high, which may cause harm to organisms, especially migratory birds.

## Methods

### Ecological risk assessment procedure based on SSDs

The SSD process was carried out according to the work of Gao *et al*.^27^ and involved (1) screening of toxicity data; (2) selection of a distribution model and fitting SSD curves; (3) calculating values for HC_5_ (hazardous concentration for 5% of species) and PNEC; and (4) describing the ecological risks. Chronic toxicity data [no observed effect concentrations (NOEC) or lowest observed effect concentrations (LOEC)] were screened from the US EPA ECOTOX database (http://cfpob.epa.gov/ecotox) following the EU technical guidance document^25^. Selected toxicity data for NP, OP, BPA, and NP2EO for freshwater are listed in the Supplementary Information Tables S1–S4, respectively. Additional toxicity data published in recent years were used that were not available for previous studies^27,30^. A sigmoid distribution was selected for constructing the SSD model. The probability distribution function and cumulative distribution function of the sigmoid distribution are shown in Supplementary Information S5.

### Calculation of HC5, PNEC_water_, and PNEC_sediment_

HC_5_ is the concentration expected to be hazardous to 5% of the species in an ecosystem^40^. According to the assessment factor methods in the EU technical guidance document^25^, to calculate the values of PNEC_water_, HC_5_ is divided by an appropriate assessment factor (AF). The AF is commonly between 1 and 5, reflecting the uncertainty of the data. In this study, the AF value was set as 3, based on the number of species tested, quantity and quality of the toxicity data, and model goodness of fit.

PNEC_sediment_ was calculated from PNEC_water_ according to the distribution equilibrium method in the EU guidance document^25^. The following formula, which is based on equilibrium partitioning theory, was applied:

1 $${{\rm{PNEC}}}_{{\rm{sediment}}}=\frac{{{\rm{K}}}_{{\rm{susp}}-{\rm{water}}}}{{{\rm{RHO}}}_{{\rm{susp}}}}\cdot {{\rm{PNEC}}}_{{\rm{water}}}$$

where K_susp-water_ is the partition coefficient of suspended matter water (L/L); RHO_susp_ is the bulk density of wet suspended matter (g/L); PNEC_water_ is the predicted no effect concentration in water (μg/L); and PNEC_sediment_ is the predicted no effect concentration in sediment (μg/g).

### Characterization of ecological risks

The risk quotient (RQ) was calculated from the ratio of predicted environmental concentrations (PEC) to PNEC values, and was used to determine the ecological risk^41^. Due to a shortage of detailed geographic distribution data for the usage and discharge amounts of PEDCs in Poyang Lake wetland, the measured environmental concentrations (MEC) were used to represent the predicted environmental concentrations. Risk levels were low, medium, or high for RQ values of <0.1, 0.1–1.0, and ≥1, respectively.

### Sampling and analysis of PEDCs

Sample collection locations were identified using a global positioning system (Fig. 1, with detailed coordinates shown in the Supplementary Information Table S6). The sampling sites were located in the Poyang Lake Wetland Park (Y25-Y28), Jiangxi Poyang Lake National Nature Reserve (Y4-Y6, Y11), five main tributaries of Poyang Lake (Y9, Y10, Y16, Y17, Y19, Y24), typical wetland lakes (Y12-Y15, Y18, Y20-Y23), typical wetlands (Y2, Y3, Y7, Y8), and the entrance of Poyang Lake into the Yangtze River (Y1). Surface water (0–1.0 m), sediment samples (0–0.15 m), and *Carex cinerascens* samples were collected from 28, 26, and 20 sites, respectively, in Poyang Lake in the dry season (October to February), according to sampling specifications.

PEDCs were extracted from the water, sediment, and *Carex cinerascens* samples by ultrasound using dichloromethane, derivatised with N, O-bis(trimethylsilyl) trifluoroacetamide. The details of the extraction, clean up, and derivatization of PEDCs in water and *Carex cinerascens* are shown in Supplementary Information S7. Sediment samples were freeze-dried using a vacuum freeze dryer (FD-1B-50, Shanghai Bilon Instrument Manufacturing Co. Ltd., Shanghai, China), and then homogenised with a stainless steel spoon, placed into pre-cleaned brown glass bottles, and finally stored at 4 °C. Sediment samples (2.0 g) were mixed in a conical bottle with 5 mL of 0.1 mol/L HCl, and pH values < 1.0 were adjusted. Next, 10 mL dichloromethane (DCM) was added, and ultrasound extraction was performed for 15 min using an ultrasonic cleaner (KQ-500E, Shanghai Bilon Instrument Manufacturing Co. Ltd.). The extraction process was repeated twice. The extracted organic phase was concentrated to approximately 3 mL. Three grams of anhydrous sodium sulfate, 4.0 g Florisil, 3.0 g anhydrous sodium sulfate, and 15–20 mL n-hexane were used to wet the pre-clean column, and the extract was poured into the column. When almost all of the extract entered the adsorption layer, it was eluted with 5.0 mL n-hexane/ether (v:v = 9:1) and 3.0 mL n-hexane/acetone (v:v = 7:3) three times. The eluent was collected and was evaporated to near-dryness using nitrogen. Fifty microliters of the derivative reagent BSTFA, 30 μL pyridine, and 20 μL 1.0 mg/L tribromophenol (internal standard, dissolved in n-hexane) were added, and then reacted for 45 min at 65 °C in a water bath. The solution was filtered to the inner column using a disposable needle filter with 0.22 μm organic membrane, to 100 μL for GC-MS analysis.

GC-MS analysis was performed with a TRACE™ 1310/TSQ8000Evo (Thermo Fisher Scientific™, Waltham, MA, USA). Separation of the target compounds was achieved using a TG-5SILMS (30 m × 0.25 mm × 0.25 μm) capillary column (Thermo Fisher Scientific™). The carrier gas was helium (99.999%) with a constant flow rate of 1 mL/min. The injector temperature was held at 260 °C, and the injection volume was 1.0 μL in splitless mode. The column temperature was programmed as follows: the initial temperature was 60 °C, increased to 150 °C at 15 °C/min, then to 220 °C at 8 °C/min, held at 220 °C for 1 min, then finally increased to 290 °C at 15 °C/min and then held at 290 °C for 5 min. The MS transfer line temperature was maintained at 280 °C, whereas the ion source temperature was 300 °C. Mass spectra were scanned in full scan mode from 50–650 m/z mass range for qualitative analysis and selected ion monitoring mode (SIM) for quantitative analysis. Electron impact ionization energy was 70 eV. All reagents used were chromatographically pure or were re-purified first. Instrumental calibration curves for NP and OP were established from standard solutions with nine concentrations ranging from 5 to 2000 μg/L. Instrument calibration curves for NP2EO and BPA were established from standard solutions with eight concentrations ranging from 5 to 1000 μg/L. All calibration coefficients were greater than 0.99, and the method quantification limits were 0.5 μg/L, 0.4 μg/L, 0.7 μg/L, and 1.2 μg/L for NP, OP, BPA, and NP2EO (RSD <5.8%, n = 5), respectively.

### Quality assurance/quality control (QA/QC)

To further validate the precision and accuracy of the method, blank and matrix spiked experiments were carried out. No PEDCs were detected in the blank samples. Mixed standard solutions of 5.0, 20.0, and 50.0 μg/L PEDCs were added (n = 6). The mean recoveries of NP, OP, BPA, and NP2EO are shown in Supplementary Information Table S8.

## Supplementary information

Supplementary Information

## Data Availability

All data generated or analysed during this study are included in this published article and its Supplementary Information.
