# Supplementary material for: Distribution and ecological risk assessment of PEDCs in the water, sediment and Carex cinerascens of Poyang Lake wetland, China
Source: Sci Rep. 2019 Aug 5;9:11302. doi: 10.1038/s41598-019-47864-4 (PMC6683139; doi:10.1038/s41598-019-47864-4)
Supplement: Supplementary file 1 — Supplementary Information [file 41598_2019_47864_MOESM1_ESM.pdf]

SUPPLEMENTARY INFORMATION

Distribution and ecological risk assessment of PEDCs in the water, sediment and *Carex cinerascens* of Poyang Lake wetland, China

Pinghua Yang[a, b *]

[a]College of Chemistry and Environment Engineering, Jiujiang University, Jiujiang 332005, China

[b]Jiangxi Province Engineering Research Center of Ecological Chemical Industry, Jiujiang 332005, China

Table S1

Chronic toxicity data of 4-nonylphenol in freshwater screened from the ECOTOX database for the SSD model.

Category	Common name (Scientific name) of species	Endpoint	Exposure Time (day)	Endpoint value (μg/L)	Reference
Algae	Green Algae (*Chara sp.)*	LOEC	4	1480	[1]
	Blue-Green Algae (*Microcystis aeruginosa)*	LOEC	12	20	[2]
	Diatom (*Melosira varians*)	NOEC	10	4	[3]
Vertebrate	Leopard Frog (*Lithobates pipiens*)	LOEC	124	10	[4]
	Wood Frog (*Lithobates sylvaticus*)	LOEC	47	10	[4]
	Goldfish (*Carassius auratus*)	LOEC	21	22	[5]
	Guppy (*Poecilia reticulata*)	NOEC	14	10	[6]
	Atlantic Salmon (*Salmo salar*)	LOEC	7	15	[7]
	Rainbow Trout (*Oncorhynchus mykiss*)	LOEC	91	10.3	[8]
	Zebra Danio (*Danio rerio*)	NOEC	5	330.5325	[9]
	Brook Stickleback (*Culaea inconstans*)	NOEC	121.76	243	[1]
	Chinese Rare Minnow (*Gobiocypris rarus*)	NOEC	28	3	[10]
	Common Carp (*Cyprinus carpio*)	LOEC	14	4	[11]
	Fathead Minnow (*Pimephales promelas*)	LOEC	42	0.33	[12]
	Medaka, High-Eyes (*Oryzias latipes*)	LOEC	14	20	[13]
	African Clawed Frog (*Xenopus laevis*)	LOEC	14	50	[14]
	Alpine Crested Newt (*Triturus carnifex*)	LOEC	30.44	19	[15]

	Black-Spotted Frog (*Pelophylax nigromaculatus*)	NOEC	15	200	[16]
	Japanese Medaka (*oryzias latipes*)	LOEC	100	9	[17]
	Channel Catfish (*Ictalurus punctatus*)	LOEC	10	60500	[18]
	Yellowfin Goby (*Acanthogobius flavimanus*)	NOEC	21	25000000	[19]
Invertebrate	Rotifer (*Trichocerca sp.*)	NOEC	20	23	[20]
	Rotifer (*Brachionus calyciflorus*)	LOEC	4	50	[21]
	Zebra Mussel (*Dreissena polymorpha*)	NOEC	112	5	[22]
	Water Flea (*Daphnia m*)	LOEC	21	245	[23]
	Midge (*Chironomus tentans*)	LOEC	3	100	[24]
	Calanoid Copepod (*Eurytemora affinis*)	NOEC	21	7	[25]
	Calanoid copepod (*Acroperus sp.*)	NOEC	20	23	[20]

Table S2

Chronic toxicity data of 4-t-octylphenol in freshwater screened from the ECOTOX database for the SSD model.

Category	Common name (Scientific name) of species	Endpoint	Exposure Time (day)	Endpoint value (μg/L)	Reference
Algae	Green Algae (*Selenastrum capricornutum*)	NOEC	4	1000	[26]
Vertebrate	Fathead Minnow (*Pimephales promelas*)	NOEC	14	9	[27]
	madaka (*Oryzias latipes*)	LOEC	60	11.4	[28]
	Rainbow Trout (*Oncorhynchus mykiss*)	LOEC	21	30	[29]
	Zebra Danio (*Danio rerio*)	LOEC	78	35	[30]
	Japanese Medaka (*Oryzias latipes*)	LOEC	60	48.1	[28]
	Wood Frog (*Lithobates sylvaticus*)	NOEC	14	206.3281	[31]
	Goldfish (*Carassius auratus*)	LOEC	2	206.3281	[32]
	Mangrove Rivulus (*Rivulus marmoratus*)	LOEC	4	300	[33]
	Leopard Frog (*Lithobates pipiens*)	LOEC	14	515.82025	[31]
	Common Carp (*Cyprinus carpio*)	LOEC	14	2500	[11]
	African Clawed Frog (*Xenopus laevis*)	LOEC	12	300000	[34]
Invertebrate	Prosobranch mollusk (*Potamopyrgus antipodarum*)	LOEC	63	5	[35]
	water flea (*Daphnia magna*)	NOEC	21	37	[36]
	Snail (*Potamopyrgus antipodarum*)	NOEC	42	100	[35]

Table S3

Chronic toxicity data of bisphenol A in freshwater screened from the ECOTOX database for the SSD model.

Category	Common name (Scientific name) of species	Endpoint	Exposure Time (day)	Endpoint value (μg/L)	Reference
Algae	Algae (*Chlorolobion braunii*)	LOEC	4	4000	[37]
	Green Algae (*Scenedesmus acutus var. acutus*)	LOEC	4	10000	[38]
Vertebrate	Brown Trout (*Salmo trutta ssp. fario*)	LOEC	30.44	1.75	[39]
	Green Swordtail (*Xiphophorus helleri*)	LOEC	60	2	[40]
	Eastern Mosquitofish (*Gambusia holbrooki*)	NOEC	7	2.295	[41]
	Chinese Rare Minnow (*Gobiocypris rarus*)	LOEC	35	15	[42]
	Goldfish (*Carassius auratus*)	LOEC	90	20	[43]
	African Clawed Frog (*Xenopus laevis*)	LOEC	14	22.82908	[44]
	Yellowfin Goby (*Acanthogobius flavimanus*)	NOEC	21	25	[45]
	Japanese Medaka (*Oryzias latipes*)	LOEC	44	117.2	[46]
	Black-Spotted Frog (*Pelophylax nigromaculatus*)	NOEC	15	200	[16]
	Zebra Danio (*Danio rerio*)	LOEC	316	400	[47]
	Crimson-Spotted Rainbowfish (*Melanotaenia fluviatilis*)	NOEC	4	500	[48]
	Oriental Weatherfish (*Misgurnus anguillicaudatus*)	LOEC	21	500	[49]
	Guppy (*Poecilia reticulata*)	NOEC	21	549	[50]
	Mangrove Rivulus (*Rivulus marmoratus*)	LOEC	4	600	[33]

	Leopard Frog (*Lithobates pipiens*)	NOEC	20	1000	[51]
	Rainbow Trout (*Oncorhynchus mykiss*)	LOEC	21	1000	[8]
	Fathead Minnow (*Pimephales promelas*)	LOEC	71	1280	[52]
	Danio (*Danio sp.*)	LOEC	6	2488.36972	[53]
	Argentine Toad (*Rhinella arenarum*)	LOEC	14	5000	[54]
	Jacopever (*Sebastes schlegelii*)	NOEC	29	100000	[55]
Invertebrate	Oligochaete, Worm (*Lumbriculus variegatus*)	LOEC	103	50	[56]
	Midge (*Chironomus riparius*)	NOEC	20	100	[57]
	European Stream Valvata (*Valvata piscinalis*)	LOEC	14	100	[58]
	Snail (*Potamopyrgus antipodarum*)	LOEC	14	100	[58]
	Snail (*Marisa cornuarietis*)	NOEC	12	100	[59]
	Amphipod (*Hyalella azteca*)	NOEC	42	490	[60]
	European Physa (*Physella acuta*)	LOEC	21	500	[61]
	Flatworm (*Dugesia japonica*)	LOEC	7	500	[62]
	Scud (*Gammarus fossarum*)	NOEC	103	500	[56]
	Scud (*Hyalella azteca*)	LOEC	42	1100	[57]
	Aquatic Sowbug (*Asellus aquaticus*)	LOEC	21	2500	[63]
	Rotifer (*Brachionus calyciflorus*)	LOEC	2	3600	[57]
	Water Flea (*Daphnia magna*)	LOEC	21	13800	[64]

Table S4

Chronic toxicity data of nonylphenol-di-ethoxylate in freshwater screened from the ECOTOX database for the SSD model.

Category	Common name (Scientific name) of species	Endpoint	Exposure Time (day)	Endpoint value (μg/L)	Reference
Algae	Green Algae (*Pseudokirchneriella subcapitata*)	LOEC	4	16000	[65]
Vertebrate	Threespine Stickleback (*Gasterosteus aculeatus*)	LOEC	21	50	[66]
	Japanese Medaka (*Oryzias latipes*)	LOEC	100	105	[17]
	Slender Tree Frog (*Litoria adelaidensis*)	LOEC	5.5833	2000	[67]
	African Clawed Frog (*Xenopus laevis*)	LOEC	4	2000	[67]
	Fathead Minnow (*Pimephales promelas*)	LOEC	6	2000	[65]
	Frog (*Crinia insignifera*)	LOEC	5.5833	4000	[67]
Invertebrate	Water Flea (*Daphnia magna*)	NOEC	6	10000	[65]
	Great Pond Snail (*Lymnaea stagnalis*)	NOEC	2	500	[68]

S5 The probability distribution function and cumulative distribution function of sigmoid distribution

The probability distribution function and cumulative distribution function of sigmoid distribution are as follows:

Three parameters: $$y = \frac{a}{1+e^{-(\frac{x-x_0}{b})}} \quad (1)$$

Four parameters: $$y = y_0 + \frac{a}{1+e^{-(\frac{x-x_0}{b})}} \quad (2)$$

Five parameters: $$y = y_0 + \frac{a}{[1+e^{-(\frac{x-x_0}{b})}]^c} \quad (3)$$

Where the y and x are the cumulative distribution function and logarithm of concentration, respectively.

Table S6

Location information of the collected samples in Poyang Lake.

Samples	Sites	Latitude	Longitude	Latitude	Longitude	Water	Sediment	*Carex cinerascens*
Y1	Hukou Ferry	29°44'57"N	116°12'17"E	29.7491667	116.2047222	+	+	+
Y2	Gutang	29°36'47.37"N	116°8'26.36"E	29.6131583	116.1406556	+	+	+
Y3	Xingzi	29°26'4.07"N	116°0'21.74"E	29.4344639	116.0060389	+	+	+
Y4	Liaonan	29°17'4.97"N	115°58'33.09"E	29.2847139	115.9758583	+	+	+
Y5	Gongqingcheng	29°11'41.95"N	115°51'12.51"E	29.1949861	115.853475	+	+	+
Y6	South Lake	29°14'31.48"N	115°49'31.38"E	29.2420778	115.8253833	+	+	+
Y7	Jishan	29°16'30"N	116°7'14"E	29.2750000	116.1205556	+	+	+
Y8	Zhouxi	29°8'13"N	116°20'52"E	29.1369444	116.3477778	+	+	+
Y9	Gan River (north)	29°11'21.27"N	116°1'26.17"E	29.1892417	116.0239361	+	+	+
Y10	Xiu River	29°11'27.45"N	116°0'42.54"E	29.1909583	116.0118167	+	+	+
Y11	Dahuchi	29°5'45.73"N	115°57'13.10"E	29.0960361	115.9536389	+	+	+
Y12	Sanlixiang	28°41'6.39"N	116°22'32.24"E	28.6851083	116.3756222	+	+	+
Y13	Junshan Lake	28°37'25.09"N	116°17'14.61"E	28.6236361	116.2873917	+	-	-
Y14	Jinxian	28°39'36.07"N	116°20'54.77"E	28.6600194	116.3485472	+	+	+
Y15	Chenjia Lake	28°39'36.07"N	116°20'54.77"E	28.6600194	116.3485472	+	-	-
Y16	Fu River	28°33'14.10"N	116°7'37.17"E	28.5539167	116.1269917	+	+	-
Y17	Fu River Tributary	28°33'25.30"N	116°7'20.80"E	28.5570278	116.1224444	+	+	+
Y18	Qinglan Lake	28°33'58.23"N	116°10'6.10"E	28.5661750	116.1683611	+	+	+
Y19	Xin River	28°41'17.75"N	116°26'14.50"E	28.6882639	116.4373611	+	+	+
Y20	Yangfang Lake	28°31'31.25"N	116°33'37.30"E	28.5253472	116.5603611	+	+	-
Y21	Dalianzi Lake	28°53'12.44"N	116°35'53.87"E	28.8867889	116.5982972	+	+	+
Y22	Kangshan Dykes (south)	28°54'53.95"N	116°28'57.83"E	28.9149861	116.4827306	+	+	-
Y23	Kangshan Dykes (north)	28°54'58.06"N	116°28'56.56"E	28.9161278	116.4823778	+	+	+
Y24	Rao River	29°7'46.77"N	116°33'25.85"E	29.1296583	116.5571806	+	+	+
Y25	Zhu Lake	29°8'40.22"N	116°44'10.04"E	29.1445055	116.7361222	+	+	-
Y26	Zhu Lake Dykes (south)	29°9'2.32"N	116°35'55.55"E	29.1506444	116.5987639	+	+	-
Y27	Zhu Lake Dykes(north)	29°9'5.94"N	116°35'56.17"E	29.1516500	116.5989361	+	+	+
Y28	Baishazhou	29°10'3.46"N	116°37'23.67"E	29.1676278	116.6232417	+	+	-

The plus sign (+) indicates that samples were collected and line segment (-) indicates that no samples were collected at this site.

S7

Extraction, clean up, and derivatization of PEDCs

1. Extraction and derivatization of PEDCs in water samples

Water samples were collected in 2 L pre-cleaned glass bottles. Methanol (1:100, v/v) was added to water samples immediately, and then the bottle was agitated by hand, in order to inhibit biological activity. Water samples were transported in boxes packed with ice and stored at 4 ℃ in a refrigerator upon arrival at the laboratory. Subsequently, water samples were filtered through a pre-combusted GF/F filter to remove particle contaminants and analyzed within 24 hours. 5 g NaCl was added to 200 mL water and 6 mol/L hydrochloric acid was added to adjust pH < 2.0, then 20 mL DCM was added, vigorously shaken, collected the organic phase by extraction method, the above extraction steps were repeated twice, merge the organic phase, dried over anhydrous Na_2SO_4 and concentrated. The extract was collected and was evaporated to near-dryness using nitrogen. Fifty microliters of the derivative reagent BSTFA, 30 μL pyridine, and 20 μL 1.0 mg/L tribromophenol (internal standard, dissolved in n-hexane) were added, and then reacted for 45 min at 65 ℃ in a water bath. The solution was filtered to the inner column using a disposable needle filter with 0.22 μm organic membrane, to 100 μL for GC-MS analysis.

2. Extraction, clean up, and derivatization of PEDCs in *Carex cinerascens* samples

Carex cinerascens samples (2.0 g) were mixed in a conical bottle with 5 mL of 0.1 mol/L HCl, and pH values <1.0 were adjusted. Next, 25 mL dichloromethane (DCM) was added, and ultrasound extraction was performed for 15 min using an ultrasonic cleaner (KQ-500E, Shanghai Bilon Instrument Manufacturing Co. Ltd.). The extraction process was repeated twice. The collected organic phase was added to 5 mL concentrated sulfuric acid in glass separating funnel, shaked vigorously, it was repeated 2-3 times until the organic phase was transparent (Dangerous! Pay attention to safety operation!). The extracted organic phase was concentrated to approximately 3 mL. Three grams of anhydrous sodium sulfate, 4.0 g Florisil, 3.0 g anhydrous sodium sulfate,

and 15-20 mL n-hexane were used to wet the pre-clean column, and the extract was poured into the column. When almost all of the extract entered the adsorption layer, it was eluted with 5.0 mL n-hexane/ether (v:v = 9:1) and 3.0 mL n-hexane/acetone (v:v = 7:3) three times. The eluent was collected and was evaporated to near-dryness using nitrogen. Fifty microliters of the derivative reagent BSTFA, 30 μL pyridine, and 20 μL 1.0 mg/L tribromophenol (internal standard, dissolved in n-hexane) were added, and then reacted for 45 min at 65 °C in a water bath. The solution was filtered to the inner column using a disposable needle filter with 0.22 μm organic membrane, to 100 μL for GC-MS analysis.

Table S8

Mean recovery and RSD of the target compounds in spiked water, sediment, and *Carex cinerascens* samples.

Compounds	Spiked level (μg/L)	Recovery (%)					
		In water	RSD(%) n=6	In sediment	RSD(%) n=6	In *Carex cinerascens*	RSD(%) n=6
NP	5.0	78.7	5.67	72.4	7.97	84.4	5.23
OP		80.6	4.77	68.9	9.12	75.7	7.10
BPA		88.8	4.81	74.8	8.62	71.3	11.05
NP2EO		79.5	4.92	80.9	7.53	75.6	6.58
NP	20.0	76.1	7.96	75.0	6.39	80.8	5.85
OP		79.1	4.97	67.0	7.90	80.0	5.49
BPA		86.8	5.51	77.3	3.98	69.8	8.72
NP2EO		78.6	4.55	83.0	7.74	73.8	7.83
NP	50.0	80.6	4.53	76.2	4.80	85.2	6.47
OP		76.3	5.42	64.6	4.73	81.6	9.23
BPA		85.3	3.98	79.5	6.70	74.3	4.22
NP2EO		77.3	6.12	86.2	6.51	73.9	4.31

Table S9

Range (mean) concentrations of 4-nonylphenol (NP), 4-t-octylphenol (OP), and bisphenol A (BPA) in surface water and sediment of China.

**Locations**	Water (ng/L)			Sediment (ng/g dw)			Reference
	NP	OP	BPA	NP	OP	BPA	
This work	0.037-21.241<br>(1.468 ±0.531)<br>n=28	0.143-7.387<br>(2.967 ±1.409)<br>n=28	3.84-242.2<br>(40.49 ±18.42)<br>n=28	0.126-17.56<br>(2.247 ±1.273)<br>n=26	0.193-5.327<br>(2.247 ±1.273)<br>n=26	1.328-38.38<br>(9.840 ±3.149)<br>n=26	
Dianchi Lake	13.6-141.6<br>n=10	nd-56.5<br>n=10	nd- 4713.6<br>n=10	-	-	-	[69]
Taihu Lake	108.7-298.5<br>(157.07)<br>n=26	21.23-305.1<br>(104.76)<br>n=26	27.95-565.40<br>(92.57)<br>n=26	nd-65.08<br>(20.66)<br>n=26	1.3-209.8<br>(38.18)<br>n=26	nd-99.2<br>(4.21)<br>n=26	[70]
Northern Taihu Lake	-	-	22.5-194(64.4)<br>n=8	-	-	6.31-291(64.1)<br>n=8	[71]
Taihu Lake	-	-	4.2-14(8.5)<br>n=23	-	-	0.19-7.4(1.3)<br>n=23	[72]
Liaohe river	-	-	5.9-141(47)<br>n=13	-	-	n.d.-0.45(0.14)<br>n=12	[72]
Hunhe river	-	-	4.4-107(40)<br>n=10	-	-	0.15-2.1(1.0)<br>n=7	[72]
Pearl River	83.3-517(292)<br>n=8	1.5-10(5.8)<br>n=8	53.7-180(107)<br>n=8	-	-	-	[73]
Pearl River Delta<br>(dry seasons)	810-3366<br>n=7	85.5-581<br>n=7	-	-	-	-	[74]
Pearl River Delta<br>(wet seasons)	36.3-390<br>n=7	2.8-22.9<br>n=7	-	-	-	-	[74]

Cape D’Aguilar Marine Reserve, Hong Kong	91.7-847.0 n=24	-	14.1-608.8 n=24	527.3-800.0 n=6	-	60.8-265.9 n=6	[75]
Fen River (wet seasons)	1.06-1036(86.3) n=29	-	-	-	-	0.04-8.46(1.82) n=29	[76]
Fen River (dry seasons)	0.63-348(37.7) n=29	-	-	-	-	1.68-48.0(10.9) n=29	[76]
Pearl River Estuaries	1740-16200	1265-15700	-	28-92	2.7-42	-	[77]
Yellow River	165.8-1187.6	2.4-4.3	-	16.6-203.8	nd-2.6	-	[78]
Huai River	215–627(421) n=12	-	23–107(62) n=12	-	-	-	[79]

dw: dry weight, nd: not detected, line segment (-) indicates no data.

Table S10

The HC_5 and $PNEC_{water}$ based on the simulated SSD curves.

Contaminants	Sample size	HC_5 (µg/L)	$PNEC_{water}$(AF=3) (µg/L)	HC_5 in literature (µg/L)	Reference	$PNEC_{water}$ in literature (µg/L)	Reference
NP	28	1.524	0.508	1.43<br>1.85	[80]<br>[81]	0.33	[84]
OP	15	1.837	0.612	1.44	[81]	0.62 (daphnids)<br>100 (algae)<br>7.7 (fish)	[74]
BPA	35	1.530	0.51	1.78	[82]	0.86	[85]
NP2EO	9	8.176	2.725	0.11	[83]		

Table S11

RQ values of 4-nonylphenol (NP), 4-t-octylphenol (OP), bisphenol A (BPA), and nonylphenol-di-ethoxylate (NP2EO) in surface water, sediment, and *Carex cinerascens* samples from the sampling sites of the Poyang Lake wetland in dry seasons (October to February).

Sites	Water				Sediment				*Carex cinerascens*			
	NP	OP	BPA	NP2EO	NP	OP	BPA	NP2EO	NP	OP	BPA	NP2EO
Y1	0.0027	0.003	0.0423	1.111	0.027	0.0144	1.102	2.036	0.0072	0.0008	0.2945	0.9834
Y2	0.0051	0.0037	0.0436	1.402	0.0204	0.0174	0.739	1.353	0.0022	0.0014	0.0487	0.51
Y3	0.0026	0.0021	0.0452	1.34	0.0354	0.0792	1.705	1.78	0.008	0.0029	0.4126	0.7944
Y4	0.0145	0.0347	0.0506	1.252	0.03	0.0228	1.351	1.726	0.029	0.0035	0.4016	0.4398
Y5	0.0007	0.0008	0.0502	0.856	0.0252	0.0936	1.13	1.555	0.0059	0.0016	0.5525	1.0158
Y6	0.0044	0.003	0.0727	1.619	0.0306	0.0318	0.695	4.48	0.0068	0.0014	0.3091	0.8688
Y7	0.0024	0.0014	0.049	0.974	0.0468	0.1176	0.274	13.09	0.0107	0.0025	0.5136	1.1496
Y8	0.0045	0.0019	0.0505	1.26	0.0474	0.0114	0.533	4.941	0.0072	0.0019	0.3663	1.0296
Y9	0.0034	0.0032	0.0451	1.327	0.0204	0.0042	0.318	2.52	0.0049	0.0269	0.2543	3.3762
Y10	0.0033	0.0035	0.0423	1.557	0.0018	0.0114	1.921	0.536	0.0031	0.0069	0.5039	1.3116
Y11	0.0023	0.0026	0.0338	1.327	0.0324	0.0162	1.673	2.429	0.0034	0.0023	0.5343	1.3998
Y12	0.0074	0.0083	0.0568	0.059	0.0366	0.036	1.173	2.371	0.0029	0.0035	0.5233	1.7112
Y13	0.0037	0.0014	0.0363	1.009	-	-	-	-	-	-	-	-
Y14	0.0026	0.0023	0.0392	1.118	0.0276	0.0252	2.606	2.236	0.0043	0.0018	0.4978	1.4322
Y15	0.0013	0.0028	0.0677	1.109	-	-	-	-	-	-	-	-
Y16	0.002	0.0026	0.0953	0.562	0.009	0.0252	1.156	1.7	-	-	-	-
Y17	0.0007	0.0023	0.0546	0.871	0.0096	0.03	0.368	2.118	0.0041	0.0067	2.114	1.4898
Y18	0.0014	0.0074	0.1312	1.642	0.003	0.0384	0.161	2.297	0.0146	0.0032	0.1241	0.5538
Y19	0.004	0.0062	0.089	1.535	0.0072	0.0234	0.181	1.292	0.0056	0.003	0.067	0.5106
Y20	0.0001	0.0018	0.3484	0.312	0.0096	0.0402	0.205	2.529	-	-	-	-
Y21	0.0008	0.0041	0.0425	0.623	0.0018	0.0246	4.671	1.837	0.0056	0.0041	0.0548	0.8676

Y22	0.0018	0.0058	0.0633	1.435	0.0156	0.027	2.275	2.084	-	-	-	-
Y23	6.00E-05	0.0002	0.0076	0.155	0.015	0.0336	1.347	2.527	0.0007	0.0032	0.0463	0.888
Y24	0.0019	0.0052	0.4751	1.646	0.0108	0.0294	0.495	2.419	0.0043	0.0051	0.14	1.6626
Y25	0.0014	0.0043	0.0346	1.056	0.003	0.033	1.513	2.639	-	-	-	-
Y26	0.001	0.0091	0.09	2.045	0.0228	0.0336	1.138	3.429	-	-	-	-
Y27	0.0011	0.0074	0.0188	1.75	0.0528	0.027	2.116	2.171	0.0031	0.0057	0.14	1.233
Y28	0.0037	0.0044	0.0482	1.166	0.2328	0.081	0.29	3.068	-	-	-	-
max	0.0145	0.0347	0.4751	2.045	0.2328	0.1176	4.671	13.09	0.029	0.0269	2.114	3.3762
min	0.00006	0.0002	0.0076	0.059	0.0018	0.0042	0.161	0.536	0.0007	0.0008	0.0463	0.4398
mean	0.00289	0.00484	0.07943	1.1471	0.02979	0.03568	1.1975	2.737	0.00668	0.00442	0.39494	1.16139
±SD	0.00105	0.00233	0.0365	0.1773	0.01688	0.0104	0.3833	0.8842	0.00286	0.0026	0.20787	0.30162

References of the Supplementary Information

[1] Liber, K., Knuth, M. L. & Stay, F. S. An integrated evaluation of the persistence and effects of 4-nonylphenol in an experimental littoral ecosystem. *Environ. Toxicol. Chem.* **18**, 357–3629 (1999).

[2] Wang, J., Xie, P. & Guo, N. Effects of nonylphenol on the growth and microcystin production of microcystis strains. *Environ. Res.* **103**, 70–78 (2007).

[3] Julius, M. L., Stepanek, J., Tedrow, O., Gamble, C. & Schoenfuss, H. L. Estrogenreceptor independent effects of two ubiquitous environmental estrogens on Melosira varians agardh, a common component of the aquatic primary production community. *Aquat. Toxicol.* **85**, 19–27 (2007).

[4] Mackenzie, C. A., Berrill, M., Metcalfe, C. & Pauli, B. D. Gonadal differentiation in frogs exposed to estrogenic and antiestrogenic compounds. *Environ. Toxicol. Chem.* **22**, 2466–2475 (2003).

[5] Soverchia, L. et al. Modulation of vitellogenin synthesis through estrogen receptor beta-1 in goldfish (Carassius auratus) juveniles exposed to 17-beta estradiol and nonylphenol. *Toxicol. Appl. Pharm.* **209**, 236–243 (2005).

[6] Li, M. H. & Wang, Z. R. Effect of nonylphenol on plasma vitellogenin of male adult guppies (Poecilia reticulata). *Environ. Toxicol.* **20**, 53–59 (2005).

[7] Lerner, D. T., Bjornsson, B. T. & McCormick, S. D. Aqueous exposure to 4-nonylphenol and 17 beta-estradiol increases stress sensitivity and disrupts ion regulatory ability of juvenile Atlantic salmon. Environ. *Toxicol. Chem.* **26**, 1433–1440 (2007).

[8] Van den Belt, K., Verheyen, R. & Witters, H. Comparison of vitellogenin responses in zebrafish and rainbow trout following exposure to environmental estrogens. *Ecotox. Environ. Safe*. **56**, 271–281 (2003).

[9] Lin, L.L. & Janz, D.M. Effects of binary mixtures of xenoestrogens on gonadal development and reproduction in zebrafish. *Aquat. Toxicol*. **80**, 382–395 (2006).

[10] Zha, J., Wang, Z., Wang, N. & Ingersoll, C. Histological alternation and vitellogenin induction in adult rare minnow (Gobiocypris rarus) after exposure to ethynylestradiol and nonylphenol. *Chemosphere*. **66**, 488–495 (2007).

[11] Huang, R. K. & Wang, C. H. The effect of two alkylphenols on vitellogenin levels in male carp. *Proceedings of the National Science Council Republic of China Part B Life Sciences.* **25(4)**, 248-252 (2001).

[12] Giesy, J. P. et al. Effects of 4-nonylphenol on fecundity and biomarkers of estrogenicity in fathead minnows (Pimephales promelas). *Environ. Toxicol. Chem*. **19**, 1368–1377 (2000).

[13] Kawana, R., Strussmann, C. A. & Hashimoto, S. Effect of p-nonylphenol on sperm motility in Japanese medaka (Oryzias latipes). *Fish Physiol. Biochem*. **28**, 213–214 (2003).

[14] Fort, D. J., Stover, E. L., Propst, T., Hull, M. A. & Bantle, J. A. Evaluation of the developmental toxicity of theophylline, dimethyluric acid, and methylxanthine metabolites using xenopus. *Drug Chem. Toxicol*. **19(4)**, 267-278 (1996).

[15] Capaldo, A. et al. Endocrine-disrupting effects of nonylphenol in the newt, triturus carnifex (amphibia, urodela). *Comp. Biochem. Physiol. Part C Toxicol. Pharmacol.* **155(2)**, 352-358 (2012).

[16] Yang, F. X., Xu, Y. & Wen, S. Endocrine-disrupting effects of nonylphenol, bisphenol A, and p,p '-DDE on rana nigromaculata tadpoles. *Bull. Environ. Contam. Toxicol*. **75(6)**, 1168 (2005).

[17] Balch, G. & Metcalfe, C. Developmental effects in japanese medaka (oryzias latipes) exposed to nonylphenol ethoxylates and their degradation products. *Chemosphere.* **62(8)**, 1214-1223 (2006).

[18] Nimrod, A. C. & Benson, W. H. Environmental estrogenic effects of alkylphenol ethoxylates. *China Environmentalence*. **26(3)**, 335-364 (1999).

[19] Mochida, K. et al. Effects of endocrine-disrupting chemicals on expression of ubiquitin c-terminal hydrolase mrna in testis and brain of the Japanese common goby. *Aquat. Toxicol.* **70(2)**, 123-136 (2004).

[20] O'Halloran, S. L., Liber, K., Gangl, J. A. & Knuth, M. L. Effects of repeated exposure to 4-nonylphenol on the zooplankton community in littoral enclosures. *Environ. Toxicol. Chem.* **18**, 376–385 (1999).

[21] Preston, B. L., Snell, T. W., Robertson, T. L. & Dingmann, B. J. Use of freshwater rotifer brachionus calyciflorus in screening assay for potential endocrine disruptors. *Environ. Toxicol. Chem.* **19(12)**, 2923-2928 (2000).

[22] Quinn, B. et al. Evaluation of the lethal and sub-lethal toxicity and potential endocrine disrupting effect of nonylphenol on the zebra mussel (Dreissena polymorpha). *Comp. Biochem. Phys. C*. **142**, 118–127 (2006).

[23] Brennan, S.J., Brougham, C.A., Roche, J.J. & Fogarty, A.M. Multi-generational effects of four selected environmental oestrogens on Daphnia magna. *Chemosphere*. **64**, 49–55 (2006).

[24] England, D. C. & Bussard, J. B. Toxicity of nonylphenol to the midge Chironomus tentans. Final Rep. 40597, 160. (ABS Lab. Inc. 1993)

[25] Forget-Leray, J., Landriau, I., Minier, C. & Leboulenger, F. Impact of endocrine toxicants on survival, development, and reproduction of the estuarine copepod Eurytemora affinis (Poppe). *Ecotox. Environ. Safe*. **60**, 288–294 (2005).

[26] ABC Laboratories, Inc. Acute Toxicity of Octylphenol to Selenastrum capricornutum Printz. Report # 31913. December. Columbia, MO, USA (1984).

[27] Brian, J. V. et al. Accurate prediction of the response of freshwater fish to a mixture of estrogenic chemicals. *Environ. Health Perspect*. **113(6)**, 721-728 (2005).

[28] Seki, M., Yokota, H., Maeda, M., Tadokoro, H. & Kobayashi, K. Effects of 4-nonylphenol and 4-tert-octylphenol on sex differentiation and vitellogenin induction in medaka (oryzias latipes). *Environ. Toxicol. Chem.* **22(7)**, 1507-1516 (2010).

[29] Belt, K. V. D., Verheyen, R. & Witters, H. Comparison of vitellogenin responses in zebrafish and rainbow trout following exposure to environmental estrogens. *Ecotoxicol. Environ. Safety*. **56(2)**, 0-281 (2003).

[30] Diel, P. et al. Comparative responses of three rat strains (da/han, sprague-dawley and wistar) to treatment with environmental estrogens. *Arch. Toxicol.* **78(4)**, 183-193 (2004).

[31] Hogan, N., Lean, D. S. & Trudeau, V. Exposures to estradiol, ethinylestradiol and octylphenol affect survival and growth of rana pipiens and rana sylvatica tadpoles. *J. Toxicol. Environ. Health Part A.* **69(16)**, 1555-1569 (2006).

[32] Isidori, M., Cangiano, M., Palermo, F. A. & Parrella, A. E-screen and vitellogenin assay for the detection of the estrogenic activity of alkylphenols and trace elements. *Comp. Biochem. Physiol. C Toxicol. Pharmacol.* **152(1)**, 51-56 (2010).

[33] Rhee, J. S., Lee, Y. M., Raisuddin, S. & Lee, J. S. Expression of r-ras oncogenes in the hermaphroditic fish kryptolebias marmoratus, exposed to endocrine disrupting chemicals. *Comp. Biochem. Physio.l C Toxicol. Pharmacol.* **149(3)**, 433-439 (2009).

[34] Huang, Y. W., Matthews, J. B., Fertuck, K. C. & Zacharewski, T. R. Use of xenopus laevis as a model for investigating in vitro and in vivo endocrine disruption in amphibians. *Environ. Toxicol. Chem.* ***24*(8)**, 2002-2009 (2010).

[35] Jobling, S. et al. Comparative responses of molluscs and fish to environmental estrogens and an estrogenic effluent. *Aquat. Toxicol.* **65(2)**, 205-220 (2004).

[36] ABC Laboratories, Inc. Chronic Toxicity of Octylphenol (4-(1,1,3,3-tetramethylbutyl)-phenol) to Daphnia magna Under Flow Through Test Conditions. Report # 36195. February. Columbia, MO, USA (1988).

[37] Gattullo, C. E., Bährs, H., Steinberg, C. E. & Loffredo, E. Removal of bisphenol A by the freshwater green alga monoraphidium braunii and the role of natural organic matter. *Sci. Total Environ.* **416(2)**, 501-506 (2012).

[38] Zhang, W. et al. Acute and chronic toxic effects of bisphenol A on chlorella pyrenoidosa and scenedesmus obliquus. *Environ. Toxicol.* **29(6)**, 714-722 (2014).

[39] Lahnsteiner, F., Berger, B., Kletzl, M., Weismann, T. Effect of bisphenol A on maturation and quality of semen and eggs in the brown trout, salmo trutta f. fario. *Aquat. Toxicol.* **75(3)**, 213-224 (2005).

[40] Kwak, H.I. et al. Effects of nonylphenol, bisphenol A, and their mixture on the viviparous swordtail fish (Xiphophorus helleri). *Environ. Toxicol. Chem.* **20**, 787-795 (2001).

[41] Scott, P. D. et al. Assessing the potential for trace organic contaminants commonly found in australian rivers to induce vitellogenin in the native rainbowfish (melanotaenia fluviatilis) and the introduced mosquitofish (gambusia holbrooki). *Aquat. Toxicol*. **185**, 105-120 (2017).

[42] Liu, Y. et al. Global and cyp19a1a gene specific dna methylation in gonads of adult rare minnow gobiocypris rarus under bisphenol a exposure. *Aquat. Toxicol.* **156**, 10-16 (2014).

[43] Hatef, A., Zare, A., Alavi, S. M. H., Habibi, H. R. & Linhart, O. Modulation of gene expression in gonad and liver of male goldfish exposed to bisphenol A. *Indian J. Sci. Technol.* **4(S8)**, 247-248 (2011).

[44] Levy, G., Lutz, I., Krüger, A. & Kloas, W. Bisphenol a induces feminization in xenopus laevis tadpoles. *Environ. Res.* **94(1)**, 0-111 (2004).

[45] Mochida, K. et al. Expression of ubiquitin c-terminal hydrolase is regulated by estradiol-17β in testis and brain of the Japanese common goby. *Fish Physiol. Biochem.* **28(1-4)**, 435-436 (2003).

[46] Sun, L. et al. Toxic effects of bisphenol A on early life stages of japanese medaka (oryzias latipes). *Bull. Environ. Contam. Toxicol.* **93(2)**, 222-227 (2014).

[47] Keiter, S. et al. Long-term effects of a binary mixture of perfluorooctane sulfonate (PFOS) and bisphenol A (BPA) in zebrafish (Danio rerio). *Aquat. Toxicol.* **118**, 116-129 (2012).

[48] Shanthanagouda, A. H., Nugegoda, D., & Patil, J. G. Effects of bisphenol a and fadrozole exposures on cyp19a1 expression in the murray rainbowfish, melanotaenia fluviatilis. *Arch. Environ. Contam. Toxicol*. **67(2)**, 270-280 (2014).

[49] Lv, X. F., Zhou, Q. F., Song, M. Y., Jiang, G. B. & Shao, J. Vitellogenic responses of 17 beta-estradiol and bisphenol a in male chinese loach (misgurnus anguillicaudatus). *Environ. Toxicol. Pharmacol.* **24(2)**, 155-159 (2007).

[50] Haubruge, E., Petit, F. & Gage, M.J. Reduced sperm counts in guppies (*Poecilia reticulata*) following exposure to low levels of tributyltin and bisphenol A. *Proc. Biol. Sci.* **267**, 2333-2337 (2000).

[51] Selcer, K. W. & Verbanic, J. D. Vitellogenin of the northern leopard frog (rana pipiens): development of an elisa assay and evaluation of induction after immersion in xenobiotic estrogens. *Chemosphere*. **112**, 348-354 (2014).

[52] Staples, C. A., Tilghman, H. A., Friederich, U., Caspers, N. & Klecka, G. M. Early life-stage and multigeneration toxicity study with bisphenol A and fathead minnows (pimephales promelas). *Ecotoxicol. Environ. Safe*. **74(6)**, 1548-1557 (2011).

[53] Selderslaghs, I. W. T., Hooyberghs, J., Blust, R. & Witters, H. E. Assessment of the developmental neurotoxicity of compounds by measuring locomotor activity in zebrafish embryos and larvae. *Neurotoxicol. Teratol.* **37**, 44-56 (2013).

[54] Wolkowicz, I. R., Herkovits, J. & Pérez Coll, C. S. Stage-dependent toxicity of bisphenol A on rhinella arenarum (anura, bufonidae) embryos and larvae. *Environ. Toxicol*. **29(2)**, 146-154 (2014).

[55] Lee, W. K. et al. Effects of environmental endocrine disruptors on the sex differentiation in korean rockfish, sebastes schlegeli. *Water Sci. Technol.* **47(9)**, 65 (2003).

[56] Ladewig, V. et al. Effects of bisphenol A on gammarus fossarum and lumbriculus variegatus in artificial indoor streams. *Toxicol. Environ. Chem. Rev*. **88(4)**, 649-664 (2006).

[57] Mihaich, E.M. et al. Acute and chronic toxicity testing of bisphenol A with aquatic invertebrates and plants. *Ecotox. Environ. Safe*. **72**, 1392-1399 (2009).

[58] Gagnaire, B. et al. Development of biomarkers of stress related to endocrine disruption in gastropods: alkali-labile phosphates, protein-bound lipids and vitellogenin-like proteins. *Aquat. Toxicol*. **92(3)**, 155-167 (2009).

[59] Schirling, M., Bohlen, A., Triebskorn, R., Köhler, H. R. An invertebrate embryo test with the apple snail marisa cornuarietis to assess effects of potential developmental and endocrine disruptors. *Chemosphere*. **64(10)**, 1730-1738 (2006).

[60] Springborn S. Bisphenol A (BPA)-chronic toxicity to amphipods (*Hyalella azteca*) under flow-through conditions. Unpublished report, No. 13796.6106 (2006).

[61] Sánchez-Argüello, P., Aparicio, N. & Fernández, C. Linking embryo toxicity with genotoxic responses in the freshwater snail *Physa acuta*: Single exposure to benzo(a)pyrene, fluoxetine, bisphenol A, vinclozolin and exposure to binary mixtures with benzo(a)pyrene. *Ecotox. Environ. Safe*. **80(3)**, 152-160 (2012).

[62] Li, M. H. Effects of bisphenol a, two synthetic and a natural estrogens on head regeneration of the freshwater planarians, dugesia japonica. *Toxicol. Environ.Chem. Rev.* **96(8)**, 1174-1184 (2014).

[63] Plahuta, M., Tišler, T., Pintar, A., & Toman, M. J. Adverse effects of bisphenol A on water louse (asellus aquaticus). *Ecotoxicol. Environ. Safety*. **117**, 81-88 (2015).

[64] Jemec, A., Tišler, T., Erjavec, B., & Pintar, A. Antioxidant responses and whole-organism changes in daphnia magna acutely and chronically exposed to endocrine disruptor bisphenol A. *Ecotoxicol. Environ. Safety*. **86(10)**, 213-218 (2012).

[65] Dorn, P. B., Salanitro, J. P., Evans, S. H. & Kravetz, L. Assessing the aquatic hazard of some branched and linear nonionic surfactants by biodegradation and toxicity. *Environ.Toxicol. Chem.* **12(10)**, 1751-1762 (2010).

[66] Sanchez, W., Palluel, O., Lagadic, L., Aã¯T-Aã¯Ssa, S. & Porcher, J. M. Biochemical effects of nonylphenol polyethoxylate adjuvant, diquat herbicide and their mixture on the three-spined stickleback (gasterosteus aculeatus l.). *Mar. Environ. Res*. **62(62 Suppl)**, S29-S33 (2006).

[67] Mann, R. M. & Bidwell, J. R. The acute toxicity of agricultural surfactants to the tadpoles of four australian and two exotic frogs. *Environ. Pollut*. **114(2)**, 195-205 (2001).

[68] Coutellec, M. A., Delous, G., Cravedi, J. P. & Lagadic, L. Effects of the mixture of diquat and a nonylphenol polyethoxylate adjuvant on fecundity and progeny early performances of the pond snail lymnaea stagnalis in laboratory bioassays and microcosms. *Chemosphere*. **73(3)**, 326-336 (2008).

[69] Yu, F., Pan, X. J., & Wang, B. Determination of four phenolic endocrine disrupting chemicals in Dianchi Lake, China. *Int. J. Environ. Anal. Chem*. **92(13)**, 1532-1545 (2012).

[70] Liu, D. et al. Occurrence, distribution, and risk assessment of alkylphenols, bisphenol A, and tetrabromobisphenol A in surface water, suspended particulate matter, and sediment in Taihu Lake and its tributaries. *Mar. Pollut. Bull.* **112(1-2)**, 142-150 (2016).

[71] Wang, Y. H., Wang, Q. Y., Hu, L. F., Lu, G. H., & Li, Y. Occurrence of estrogens in water, sediment and biota and their ecological risk in northern taihu lake in china. *Environ. Geochem. Health*. **37(1)**, 147-156 (2015).

[72] Jin, H. B. & Zhu, L. Y. Occurrence and partitioning of bisphenol analogues in water and sediment from Liaohe River Basin and Taihu Lake, China. *Water Res*. **103**, 343-351 (2016).

[73] Gong, J., Huang, Y. D., Huang, W., Ran, Y., & Chen, D. Y. Multiphase partitioning and risk assessment of endocrine-disrupting chemicals in the Pearl River, China. *Environ. Toxicol. Chem.* **35(10)**, 2474-2482 (2016).

[74] Chen, R. et al. Spatial–temporal distribution and potential ecological risk assessment of nonylphenol and octylphenol in riverine outlets of pearl river delta, china. *J. Environ. Sci.* **26(11)**, 2340-2347 (2014).

[75] Xu, E. G. B., Morton, B., Lee, J. H. W., & Leung, K. M.Y. Environmental fate and ecological risks of nonylphenols and bisphenol A in the Cape D'Aguilar Marine Reserve, Hong Kong. *Mar. Pollut. Bull.* **91(1)**, 128-138 (2015).

[76] Liu, X. W. et al. Distributions and ecological risk assessment of estrogens and bisphenol A in an arid and semiarid area in northwest China. *Environ. Sci. Pollut. Res.* **24(8)**, 7216-7225 (2017).

[77] Zhong, M. Q., Yin, P. H., & Zhao, L. Nonylphenol and octylphenol in riverine waters and surface sediments of the Pearl River Estuaries, South China: occurrence, ecological and human health risks. *Water Sci. Technol.-Water Supply*. **17(4)**, 1070-1079 (2017).

[78] Wang, L. et al. Monitoring of selected estrogenic compounds and estrogenic activity in surface water and sediment of the Yellow River in China using combined chemical and biological tools. *Environ. Pollut*. **165**, 241–249 (2012).

[79] Niu, S. P. & Zhang, C. L. Endocrine Disrupting Compounds from the Source Water of the Huai River (Huainan City), China. *Arch. Environ. Contam. Toxicol*. **74(3)**, 471-483 (2018).

[80] Gao, P., Li, Z. Y., Gibson, M., & Gao, H. W. Ecological risk assessment of nonylphenol in coastal waters of china based on species sensitivity distribution model. *Chemosphere*. **104(3)**, 113-119 (2014).

[81] Chen, R. Distribution characteristics and risk assessment of nonylphenol and octylphenol in water and sediments from riverine runoff of the Pearl River Delta. Master dissertation, Jinan University (2014).

[82] Liu, C. Distribution characteristics and ecological risk assessment of typical phenolic endocrine disrupting chemicals in Daliao River estuary. Master dissertation, Ocean University of China (2012).

[83] Fenner, K., Kooijman, C., Scheringer, M., & Hungerbühler, K. Including transformation products into the risk assessment for chemicals: the case of nonylphenol ethoxylate usage in Switzerland. *Environ. Sci. Technol.* **36(6)**, 1147-1154 (2002).

[84] ECB (European Chemicals Bureau). European Union risk-assessment report for 4-Nonylphenol (branched) and Nonylphenol. European Commission Joint Research Centre, Ispra, Italy (2001).

[85] Guo, L., Li, Z. Y., Gao, P., Hu, H. & Gibson, M. Ecological risk assessment of bisphenol A in surface waters of China based on both traditional and reproductive endpoints. *Chemosphere.* **139**, 133-137 (2015).
